# Root Canal Filling of Teeth with Open Apex Using Different Techniques, Low-Temperature Gutta-Percha Points, and Bioceramic Sealers

**DOI:** 10.1590/0103-644020267025

**Published:** 2026-04-10

**Authors:** Rui Pereira da Costa, Igor Bassi Ferreira Petean, Guilherme Nilson Alves dos Santos, Alice Corrêa Silva-Sousa, Rafael Verardino Camargo, Fabiane Carneiro Lopes-Olhê, Jardel Francisco Mazzi-Chaves, Renato Roperto, Yara Teresinha Corrêa Silva-Sousa, Manoel Damião Sousa-Neto

**Affiliations:** 1 Department of Restorative Dentistry, School of Dentistry of Ribeirão Preto, University of São Paulo (USP), Ribeirão Preto, SP, Brazil; 2 Bitonte College of Dentistry, Northeast Ohio Medical University, Ohio, Estados Unidos; 3 Department of Restorative Dentistry, School of Dentistry of Ribeirão Preto, University of São Paulo (USP), Ribeirão Preto, SP, Brazil

**Keywords:** push-out, gutta-percha, filling, bioceramic, open apex

## Abstract

To evaluate the impact of different root canal filling techniques on the push-out resistance to dislodgment of the filling material to dentin, the quality of the filling interface, and root temperature variation in teeth with open apex. Forty-eight human maxillary canines with open apex were divided into 4 experimental groups, according to the technique for root canal filling: Group I - apical barrier with MTA and backfill technique, Group II - apical filling with conventional points and continuous wave technique (4mm/200ºC), Group III - apical filling with low-fusion gutta-percha points and continuous wave technique (4mm/100ºC), Group IV - conventional single cone technique. Temperature changes on the outer surface of the root were measured in thirds during the filling. BS was evaluated using the push-out test. The adhesive interface was examined using scanning electron microscopy. Analysis of variance was used to analyze BS and temperature variation data, while the chi-square test was used to analyze failure type. The fit of the filling material to the dentin wall was evaluated using non-parametric Mann-Whitney and Kruskal-Wallis tests, followed by Dunn’s test. The group using the MTA barrier (4.5±3.5) showed higher BS values compared to the conventional points (2.8±1.1), low-fusion points (3.1±2.1), and the single cone technique (2.7±2.3) (p<0.05). In terms of temperature variation, a greater temperature increase was observed in the low-fusion points group (p<0.05). MTA barrier exhibited the highest BS values and adhesive interface quality, while the continuous wave condensation technique, regardless of the type of gutta-percha point used, showed intermediate values compared to the single cone technique.

## Resumo

Avaliar o impacto de diferentes técnicas de obturação do canal radicular na resistência de união ao deslocamento (*push-out*) do material obturador à dentina, na qualidade da interface de preenchimento e na variação da temperatura radicular em dentes com ápice aberto. Quarenta e oito caninos superiores humanos com ápice aberto foram divididos em 4 grupos experimentais, de acordo com a técnica de obturação do canal radicular: Grupo I - barreira apical com MTA e técnica de*backfill*; Grupo II - obturação apical com cones convencionais e técnica de onda contínua (4mm/200°C); Grupo III - obturação apical com cones de guta-percha de baixa fusão e técnica de onda contínua (4mm/100°C); Grupo IV - técnica de cone único convencional. As alterações de temperatura na superfície externa da raiz foram medidas em terços durante a obturação. A resistência de união (RU) foi avaliada pelo teste de*push-out*. A interface adesiva foi examinada por microscopia eletrônica de varredura. A análise de variância foi utilizada para analisar os dados de RU e de variação de temperatura, enquanto o teste qui-quadrado foi utilizado para analisar o tipo de falha. O ajuste do material obturador à parede dentinária foi avaliado por meio dos testes não paramétricos de Mann-Whitney e de Kruskal-Wallis, seguidos do teste de Dunn. O grupo que utilizou a barreira de MTA (4,5±3,5) apresentou valores de RU superiores em comparação com os cones convencionais (2,8±1,1), os cones de baixa fusão (3,1±2,1) e a técnica de cone único (2,7±2,3) (p<0,05). Em termos de variação de temperatura, observou-se maior aumento de temperatura no grupo de cones de baixa fusão (p<0,05). A barreira de MTA exibiu os maiores valores de RU e de qualidade da interface adesiva, enquanto a técnica de condensação por onda contínua, independentemente do tipo de cone de guta-percha utilizado, apresentou valores intermediários em comparação à técnica de cone único.I remain at your disposal for any further information.

**Figure:**
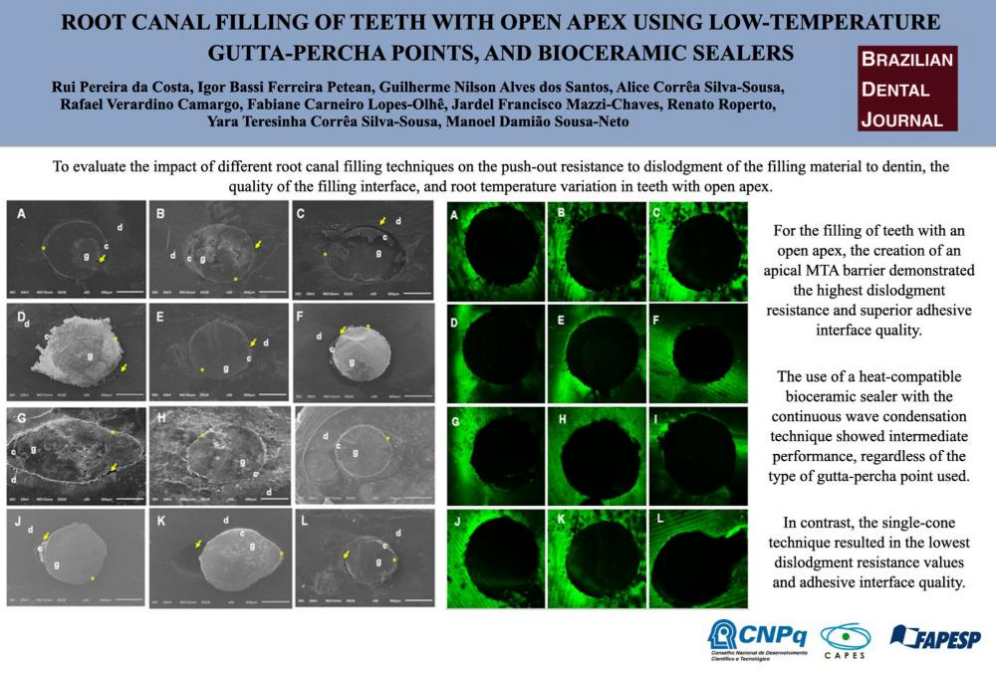

## Introduction

Traumatic dental injuries, particularly those involving developing teeth, often result in immature roots characterized by an open apex, wide root canals, and thin dentinal walls^1^. These injuries can cause various complications, starting with pulp necrosis and progressing to apical periodontitis^1^. Typically, these cases require root canal treatment, which poses significant challenges in their clinical management, mainly due to the open apex.

When obturating root canals with an open apex, there is a frequent risk of overfilling and overextension due to the lack of an apical constriction^2^. This can lead to the extrusion of filling materials ^2^ ^,^ ^3^. Therefore, two factors need to be considered when planning the filling of root canals with an open apex: the choice of filling material, which must promote three-dimensional sealing, especially in the apical region, and the filling technique, which should allow for control of the material to prevent leakage into the periapical tissues^4^.

For filling teeth with an open apex, it is recommended to use a calcium hydroxide-based intracanal paste beforehand. The purpose of applying calcium hydroxide multiple times is to create a mineralized barrier strong enough to support traditional filling with gutta-percha and endodontic sealer^5^ ^,^ ^6^ ^,^ ^7^. However, it is important to emphasize that this technique has limitations, such as the unpredictability of mineralized barrier formation, the necessity for frequent follow-up appointments to adjust the medication, and the increased risk of root fracture during the treatment period^5^ ^,^ ^6^ ^,^ ^7^.

The introduction of an apical plug with mineral trioxide aggregate (MTA) aimed to establish a physical barrier of at least 4 mm in thickness to achieve complete apical sealing^8^ ^,^ ^9^. Additionally, the material's bioactivity is expected to promote apexification by virtue of its sealing properties, biocompatibility, and ability to induce the formation of mineralized tissue^8^ ^,^ ^9^. This technique has become the gold standard for apexification in a single session^1^ ^,^ ^10^ ^,^ ^1^.

Another option for achieving apical sealing in teeth with an open apex is to use bioceramic sealers. These sealers have bioactive potential due to the release of calcium ions^12^ ^,^ ^13^ and the ability to induce the mineralization process for mineralized barrier formation^14^. Additionally, these materials chemically adhere to dentin and expand upon setting, thereby enhancing apical sealing^15^. The new generation of bioceramic sealers enables their use in thermoplastic filling techniques as they can endure temperature variations without undergoing changes in their physical-chemical properties^16^ ^,^ ^17^.

Filling wide root canals in a three-dimensional manner presents a challenge. Thermoplastic filling techniques, such as the continuous wave condensation technique, can help address this challenge. This technique involves achieving vertical condensation in the apical portion (Downpack) by employing heat carriers set at temperatures between 200 and 220 °C, allowing penetration up to 4 mm from the apical foramen^18^. However, it is important to note that the increase in temperature inside the root canal can have a negative impact on the periapical tissues, especially in cases of teeth with an open apex, as they are more susceptible to filling material leakage^4^ ^,^ ^18^. The development of low-fusion gutta-percha points, produced by injection molding with Conform Fit technology, allows the continuous wave condensation technique to be performed at a reduced temperature of 100 °C^18^. This minimizes damage to periapical tissues. Additionally, the higher rate of heat transmission reported by the manufacturer allows the use of heat carriers at a greater distance from the apical foramen without compromising the quality of filling in the apical third of teeth^18^.

Currently, there is no scientific evidence validating the quality of fillings done with low-fusion gutta-percha points combined with new bioceramic sealers used in the continuous condensation wave technique with modification of the Downpack temperature. This study aims to assess the push-out resistance to dislodgment of the filling material to dentin, the quality of the filling interface, and the temperature variations observed during each technique, achieved using the continuous wave condensation technique with bioceramic sealer employing both conventional and low-fusion gutta-percha points, as well as the single cone technique, in comparison with the technique of creating an apical barrier with MTA in teeth with an open apex. The null hypothesis stated that there would be no significant differences among the evaluated protocols regarding^1^ the push-out resistance to dislodgment of the filling material to dentin and^2^ the quality of the adhesive interface.

## Materials and Methods

This study was approved by the local Research Ethics Committee. The sample size calculation was performed using SigmaPlot v.12.00 software (Systat Software, San Jose, CA), based on parameters derived from previous studies^19^ ^,^ ^20^ ^,^ ^21^ ^,^ ^22^ ^,^ ^23^. A significance level of α = 0.05 and a statistical power of 0.9 were adopted, resulting in an estimated minimum of 10 specimens per group for the push-out tests, failure pattern assessment, and adhesive interface analysis.

Forty-eight extracted human maxillary canines with a minimum root length of 16 mm and a major-to-minor diameter ratio between 1.5 and 2.0 were selected. These parameters were confirmed by scanning using a PreXion 3D® cone beam computed tomography unit (Prexion Co. Ltd, Tokyo, Japan)^19^ ^,^ ^20^ ^,^ ^21^ ^,^ ^22^ ^,^ ^23^. The acquisition protocol included 90 kV, 4 mA, and 37 seconds of exposure, with an isotropic voxel size of 0.10 mm and a field of view measuring 5×5 mm^19^ ^,^ ^20^ ^,^ ^21^ ^,^ ^22^ ^,^ ^23^.

Following sample selection, the tooth crowns were sectioned, and the roots were standardized to a length of 16 mm using a precision saw (Isomet 1000, Buehler, Lake Bluff, IL, USA)^18^. The root canals were then irrigated with a 2.5% sodium hypochlorite (NaOCl) solution using a disposable plastic syringe (Ultradent Products Inc., South Jordan, UT, USA) and a 30G NaviTip needle (Ultradent Products Inc., South Jordan, UT, USA)^18^.

The canals were initially explored with a #15 K-file (FKG Dentaire, La Chaux-de-Fonds, Switzerland). After irrigation with 2 mL of 2.5% NaOCl, all specimens were instrumented using the ProTaper Ultimate system (Dentsply Sirona, Ballaigues, Switzerland). Instrumentation was performed up to #35/.12 file 3 mm beyond the apical foramen to create an open apex with an approximate diameter of ISO #70, followed by preparation to the FXL file (#50/.10) positioned 1.0 mm short of the root apex to standardize the canal dimensions^18^ ^,^ ^19^ ^,^ ^20^ ^,^ ^21^ ^,^ ^22^ ^,^ ^23^.

After preparation, canals were irrigated with 17% EDTA for 3 minutes, followed by a final flush with 1 mL of 2.5% NaOCl, and then dried. Subsequently, specimens were randomly assigned using the random.org platform (http://www.random.org) into four experimental groups (n=12 per group), according to the filling techniques and materials applied (Table I). In each group, ten teeth were filled with bioceramic cement, while two specimens received bioceramic cement mixed with a fluorescent dye for confocal laser scanning microscopy (CLSM) evaluation.

**Table I:** Endodontic materials, gutta-percha cones and filling sealer that used in the experimental procedures, with their respective trade names, manufacturer and composition, according to information provided by the manufacturer.

Material	Manufacturer	Composition
MTA	Angelus, Londrina, Paraná	Tricalcium silicate, dicalcium silicate, tricalcium aluminate, calcium oxide, tetracalcium ferroaluminate, bismuth oxide
Conform Fit Gutta-Percha ProTaper Ultimate	Dentsply De Trey GmbH, Konstanz, Germany	Synthetic Gutta-Percha (20-30%), Zinc Oxide (45-55%), Titanium Dioxide (1-10%), Barium Sulfate (15-20%), Inert Fillers (<10%), Yellow Dye (<2%), Red Dye (<2%)
AutoFit Greater Taper Gutta Percha Points	Kerr Corporation, Kloten, Switzerland	Zinc Oxide (60-100%)
VDW.1 Bioceramic Sealer	Dentsply De Trey GmbH, Konstanz, Germany	Zirconium dioxide, tricalcium silicate, dimethyl sulfoxide, lithium carbonate and thickening agents.

Group I - Apical barrier with MTA and Backfill technique: The apical barrier was created by placing successive increments of MTA (MTA Branco, Angelus, Londrina, PR, Brazil), mixed at a 1:1 powder-to-liquid ratio, using paper points and vertical condensers until a thickness of 4 mm was achieved. After creating the apical barrier, the root canal walls were coated with VDW.1SEAL bioceramic endodontic sealer (Dentsply Sirona, Ballaigues, Switzerland) using a lentule spiral. Then, the middle and cervical thirds of the canal were gradually filled (Backfill) with injectable gutta-percha using the Flow handpiece of the Gutta-Smart device.

Group II - Conventional point and continuous wave technique: A conventional gutta-percha point (Auto Fit Greater Taper, Kerr Corporation, Kloten, Switzerland) calibrated up to diameter #70 using a calibrating ruler (Dentsply Maillefer, Ballaigues, Switzerland) was used. The adaptation of the gutta-percha point to the working length (WL) was verified by tactile assessment of the apical stop and resistance to removal (tug-back), as well as by taking digital radiographs in the ortho and mesioradial directions. This procedure was performed in preparation for obturation with the continuous wave condensation technique. The gutta-percha point was coated with VDW.1SEAL bioceramic endodontic sealer and introduced in a circular and gradual movement up to the WL. A heat carrier (Downpack) connected to the Pack handpiece of the Gutta-Smart device (Dentsply Sirona, Ballaigues, Switzerland) was activated to penetrate the gutta-percha point. The temperature was set at 200 ºC until it reached a distance of 4 mm short of the WL. After applying 10 seconds of apical pressure with the heat deactivated, the temperature was reactivated for 1 second to detach the gutta-percha from the carrier, which was then removed. Subsequently, vertical condensation was performed. The middle and cervical thirds were incrementally filled as described in Group I. 

Group III - A low-fusion gutta-percha point (ProTaper Ultimate Conform Fit FX, Dentsply Sirona, Ballaigues, Switzerland), calibrated to ISO #70, was positioned 4 mm short of the working length. The point was coated with VDW.1SEAL bioceramic endodontic sealer and then confirmed for its adaptation at the working length. Subsequently, a heat carrier (Downpack) connected to the Pack handpiece of the Gutta-Smart heat transfer device was used to penetrate the gutta-percha at a temperature of 100ºC. After applying apical pressure and triggering the temperature for 1 second to separate the gutta-percha from the heat carrier, vertical condensation was performed, and the middle and cervical thirds were incrementally filled as described in Group I.

Group IV - Single cone technique: the teeth were filled using the single-cone technique with a low-fusion gutta-percha point (Conform Fit FX) calibrated to a #70 diameter using a measuring ruler. After placement, adaptation, and sealer coating of the cone were verified as described in Group II. Any excess filling material was then removed using a heated Hollemback instrument (Golgran, Sao Caetano do Sul, Brazil). Subsequently, cold vertical condensation was performed by applying light apical pressure with a Paiva condenser (Golgran, São Caetano do Sul, Brazil) while the gutta-percha remained plasticized.

The endodontic access on each specimen in all groups was sealed with temporary restorative material (Ketac Molar EasyMix; 3M, Maplewood, MN, USA). The specimens were then placed in an oven at 37°C in a 100% humidity environment (using gauze moistened with distilled water). 

Two other teeth from each group were filled with bioceramic sealer plus the fluorescent dye Calcein-AM (Sigma-Aldrich, Merck KGaA, Darmstadt, Germany) using the specific filling technique for each experimental group, for analysis in CLSM. 

### Temperature analysis during filling techniques

During the filling procedures, three type K thermocouples (MINIPA, São Paulo, Brazil) connected to digital thermometers were used. To ensure constant and firm contact between the sensors and the external root surface, an adjustable acrylic^18^ device was employed to stabilize the thermocouple wires against the root surface at 15 mm, 7 mm, and 3 mm from the root apex within the device^18^. This configuration enabled the measurement of maximum and minimum temperatures on the external root surfaces of the cervical, middle, and apical thirds^18^. The ambient temperature was maintained at 26°C (± 0.5°C) throughout the experiment^18^.

### Push-out test and failure pattern analysis

The roots were cut into 1.0 mm (± 0.2 mm) thick slices using an Isomet 1000 cutting machine (Buehler, Lake Forest, IL, USA) after the endodontic sealer had set for a period equal to three times its standard setting duration^18^. This resulted in nine slices per root.

Two slices from each third of the material were placed on stainless steel bases in the lower part of the Model 2519-106 universal testing machine (Instron Inc., Canton, MA, USA) ^18^ ^,^ ^19^ ^,^ ^20^ ^,^ ^21^ ^,^ ^22^ ^,^ ^23^. This was done in accordance with the diameter of the root canal and metal rods^18^ ^,^ ^19^ ^,^ ^20^ ^,^ ^21^ ^,^ ^22^ ^,^ ^23^. The root slices were positioned with the root canal aligned with the orifice in the metal base, and their cervical surface facing downwards^18^ ^,^ ^19^ ^,^ ^20^ ^,^ ^21^ ^,^ ^22^ ^,^ ^23^. The plungers were secured to the upper crosshead of the testing machine and positioned in contact with the filling material^18^ ^,^ ^19^ ^,^ ^20^ ^,^ ^21^ ^,^ ^22^ ^,^ ^23^. The testing machine was operated at a constant crosshead speed of 0.5 mm/min until the maximum stress required to displace the filling material was reached^18^ ^,^ ^19^ ^,^ ^20^ ^,^ ^21^ ^,^ ^22^ ^,^ ^23^.

The force required to displace the filling material was recorded in Newtons (N) ^18^ ^,^ ^19^ ^,^ ^20^ ^,^ ^21^ ^,^ ^22^ ^,^ ^23^. To calculate the push-out resistance to dislodgment in Megapascals (MPa), this force was divided by the lateral surface area of the filling material^18^ ^,^ ^19^ ^,^ ^20^ ^,^ ^21^ ^,^ ^22^ ^,^ ^23^. The height (h) of each slice was measured with a digital caliper, while the major (R) and minor (r) radii were assessed using a stereomicroscope (Leica M165C; Leica Microsystems, Mannheim, Germany) and LAS v.4.4 software^18^ ^,^ ^19^ ^,^ ^20^ ^,^ ^21^ ^,^ ^22^ ^,^ ^23^. 

To analyze the failure pattern, the previously sectioned slices were examined from the cervical view both before and after the push-out test. The analysis was performed using a Leica stereomicroscope (Leica Mycrosystems, Mannheim, Germany) at 25x magnification, and the LAS v4.4 software program (Leica Mycrosystems, Mannheim, Germany)^18^ ^,^ ^19^ ^,^ ^20^ ^,^ ^21^ ^,^ ^22^ ^,^ ^23^. Failure modes were classified after the push-out test as follows: (a) adhesive to dentin, if the filling material detached from the dentin; (b) adhesive to sealer, if the gutta-percha detached from the sealer; (c) mixed, if detachment occurred at both interfaces; (d) cohesive in dentin, if fracture occurred within the dentin; and (e) cohesive in sealer, if fracture occurred within the sealer^18^.

### Qualitative-quantitative analyses of the interface by Scanning Electron Microscopy (SEM).

For qualitative analysis of the adhesive interface by SEM, the third slice obtained from each root third was used. Slices were prepared by decalcification in 6 M hydrochloric acid (HCl), deproteinization in 2% sodium hypochlorite (NaOCl), and dehydration following protocols described in recent studies (19-23). Following vacuum sputter-coating, specimens were examined in a scanning electron microscope (model JSM 5410, JEOL Ltd., Tokyo, Japan) operated at 20 kV^18^ ^,^ ^19^ ^,^ ^20^ ^,^ ^21^ ^,^ ^22^ ^,^ ^23^.

Images were acquired at 100×, 250×, and 500× magnifications. For the 250× images, twelve measurements were obtained at equally spaced points along the adhesive interface to detect any voids ^18^ ^,^ ^19^ ^,^ ^20^ ^,^ ^21^ ^,^ ^22^ ^,^ ^23^. Two trained examiners independently evaluated the images in a double-blind design, and intra-examiner agreement was assessed using the Kappa index. The agreement between the examiners was found to be excellent (0.98).

According to the method described by Balguerie et al^24^, the adaptation of the sealer to the canal wall was classified using the following criteria: a) Good: most sections exhibited no gaps between the sealer and dentin; b) Reasonable: most sections displayed minor flaws (<1 µm) at the interface; c) Poor: most sections showed numerous gaps (1-10 µm) between sealer and dentin; d) No adaptation: most sections exhibited gaps >10 µm, indicating no adaptation^20^ ^,^ ^21^ ^,^ ^23^.

### Qualitative analysis of the adhesive interface by Confocal Laser Scanning Microscopy (CLSM)

Qualitative analysis of the adhesive interface was conducted on the cervical surfaces of the third slice from each root third using confocal laser scanning microscopy (CLSM)^18^. 

The slices for CLSM were prepared by polishing, washing in an ultrasonic tank, and then conditioning with 17% EDTA. Slices were subsequently rinsed and dried with absorbent paper according to protocols described in recent studies.^20^ ^,^ ^21^ ^,^ ^23^. These slices were then placed in the CLSM (LEXT OLS4000®, Olympus Corporation, Shinjuku, Tokyo, Japan) to capture images of the adhesive interface using the OLS4100 software (Olympus Corporation, Shinjuku, Tokyo, Japan) at magnifications of 5x, 20x, and 50x. Representative images of each quadrant were also captured at 20× magnification.

### Qualitative analysis of sealer penetration by Confocal laser scanning fluorescence microscopy (CLSFM)

In specimens obturated with bioceramic sealer combined with the fluorescent dye Calcein-AM, qualitative analysis of sealer penetration was performed^18^. Three slices of each third were analyzed at a depth of 10 μm below the sample surface using objective lenses at 10x, 20x, 50x, and 100x magnifications (Leica Application Suite-Advanced Fluorescence, Leica Systems) (da Costa et al., 2024). Analysis was performed in a 5×5 cm field of view at a resolution of 512×512 pixels, using epifluorescence mode with excitation/emission wavelengths of 360/449 nm for calcein^18^.

### Statistical Analysis

Analysis of variance (two-way ANOVA) followed by Tukey’s test was applied to assess temperature variations and push-out resistance to dislodgment among the experimental groups. Data were further stratified by root third (cervical, middle, and apical) for analysis. The chi-square test was used to evaluate the type of failure after the push-out resistance to dislodgment test. Additionally, non-parametric tests, including the Mann-Whitney and Kruskal-Wallis tests (p<0.05), followed by Dunn’s test for multiple comparisons, were used to analyze the adaptation of the filling material to dentin in SEM images.

## Results

Table II shows the mean and standard deviation values of dislodgment resistance for different filling protocols: MTA, conventional, and low-fusion gutta-percha points with the use of the continuous wave condensation technique, as well as low-fusion points with the single cone technique. The analysis of variance indicated significant differences among the groups (p<0.05). The Tukey test demonstrated that the MTA group (4.5 ± 3.5) exhibited higher dislodgment resistance values compared to the conventional points with continuous wave technique (2.8 ± 1.1), the low-fusion points with continuous wave technique (3.1 ± 2.1), and the single-cone technique (2.7 ± 2.3). The latter three protocols showed no significant differences among them (p>0.05).

Comparison of the different filling techniques across the cervical, middle, and apical thirds of the root canal revealed statistically significant differences. The Tukey test indicated that in the MTA group, dislodgment resistance was higher in the apical third (8.4 ± 3.1) (p<0.05) compared to the cervical (3.0 ± 1.6) and middle (2.1 ± 1.3) thirds, which did not differ significantly from each other (p>0.05). In the conventional point and low-fusion point groups using the continuous wave technique, no significant differences were found between root thirds (p>0.05). Conversely, in the single-cone technique group, the apical third showed lower dislodgment resistance values (1.3 ± 1.2) compared to the cervical (3.9 ± 2.5) and middle thirds (3.0 ± 2.2) (p<0.05) (Table III).

**Table II:** Mean and standard deviation values, in megapascals (MPa), of push-out resistance to dislodgment according to filling techniques and gutta-percha cones used in each group.

	Mean ± SD
MTA	4.5 ± 3.5A
Conventional Cone *Downpack at* 4 mm.	2.8 ± 1.1 B
Low fusion cone *Downpack* at 4 mm	3.1 ± 2.1 B
Single Low fusion cone	2.7 ± 2.3 B

Different capital letters indicate statistical differences in the lines. Tukey Test (P< 0.05).

**Table III:** Mean and standard deviation values, in megapascals (MPa), of push-out resistance to dislodgment according to filling techniques and gutta-percha cones used in each group.

	Cervical	Middle	Apical
MTA	3.0±1.6 Ba	2.1±1.3 Ba	8.4 ± 3.1 Aa
Conventional Cone *Downpack at* 4 mm	3.0 ± 1.0 Aa	2.1 ± 0.8 Aa	3.2± 1.3 Ab
Low fusion cone *Downpack* at 4 mm	3.3 ± 1.3 Aa	2.5 ± 2.1 Aa	3.3± 2.6 Ab
Single Low fusion cone	3.9 ± 2.5 Aa	3.0 ± 2.2 Aa	1.3± 1.2 Bc

Different capital letters indicate statistical difference in rows and lowercase letters indicate statistical difference in the lines. Tukey Test (P< 0.05).

Table IV presents the distribution of failure patterns. The chi-square test indicated statistically significant differences among the groups (p<0.05). In the MTA group, mixed and cohesive failures in dentin predominated. The conventional point with the continuous wave technique exhibited mainly adhesive failures to the filling material and mixed failures. The low-fusion-point group with the continuous wave technique showed a predominance of mixed failures. The single-cone technique group presented mostly adhesive failures to dentin and to the filling material (Table IV).

**Table IV:** Type of failure in percentage after the push-out test according to filling techniques and gutta-percha cones used in each group, in the different thirds of the root canal.

	MTA			Conventional Cone Downpack at 4 mm			Low fusion cone Downpack at 4 mm			Single Low fusion cone		
	C	M	A	C	M	A	C	M	A	C	M	A
Ad	10	0	0	10	20	30	0	0	0	80	30	60
Amo	0	0	0	50	70	30	10	10	20	10	50	30
M	90	100	0	40	10	40	90	90	80	10	20	10
Cd	0	0	100	0	0	0	0	0	0	0	0	0

Types of failure Ad = Adhesive to dentin; Amo = Adhesive to filling material; M = mixed; Cd=Cohesive in dentin;

Root Canal thirds: C= Cervical third; M= Middle third; A= Apical third.

Table V presents the SEM scores. Non-parametric Mann-Whitney and Kruskal-Wallis tests indicated that the conventional point and low-fusion point groups using the continuous wave technique exhibited a higher frequency of good and reasonable adaptation (gaps between 1 μm and 10 μm) compared to the single-cone technique (p<0.05). No significant differences were found among the cervical, middle, and apical thirds (p>0.05).

**Table V:** Percentage distribution of the types of filling material adaptation to root dentin, evaluated by means of SEM in accordance with the filling techniques and gutta-percha cones used in each group.

	MTA			Conventional Cone Downpack at 4 mm			Low fusion cone Downpack at 4 mm			Single Low fusion cone		
	C	M	A	C	M	A	C	M	A	C	M	A
Good	0	33.3	66.7	33.3	33.3	66.7	0	0	66.7	0	0	0
Reasonable	100	66.7	33.3	66.7	66.7	33.3	100	100	33.7	33.3	0	66.7
Poor	0	0	0	0	0	0	0	0	0	33.3	33.3	33.3
S/A	0	0	0	0	0	0	0	0	0	33.4	66.7	0

Root Canal thirds: C= Cervical third; M= Middle third; A= Apical third.

Analysis of temperature variation during obturation showed that in the MTA barrier, conventional points with the continuous wave technique, and low-fusion points with the continuous wave technique groups, lower temperature variations were recorded in the apical third compared to the cervical and middle thirds (p<0.05), which were similar to each other (p>0.05). In contrast, no statistically significant differences in temperature variation were observed among root thirds in the single-cone technique group (p>0.05). Table VIpresents the temperature variation data (°C) for each technique.

**Table VI:** Mean values and standard deviation of temperature variation during endodontic treatment in different root thirds.

	Cervical	Middle	Apical
MTA	1.6 ± 1.0Ba	0.9 ± 0.8Ba	0.3 ± 0.4Bb
Conventional Cone Downpack at 4 mm	4.3 ± 1.7ABa	6.2 ± 3.4Aa	3.0 ± 1.2Ab
Low fusion cone Downpack at 4 mm	8.5 ± 3.8Aa	9.5 ± 3.6Aa	4.6 ± 2.4Ab
Single Low fusion cone	0.2 ± 0.5Ba	0.3 ± 0.2Ba	0.1 ± 0.2Ba

*Different capital letters indicate statistical difference in rows and lowercase letters indicate statistical difference in the lines. Tukey Test (P< 0.05).

**Figure 1:**
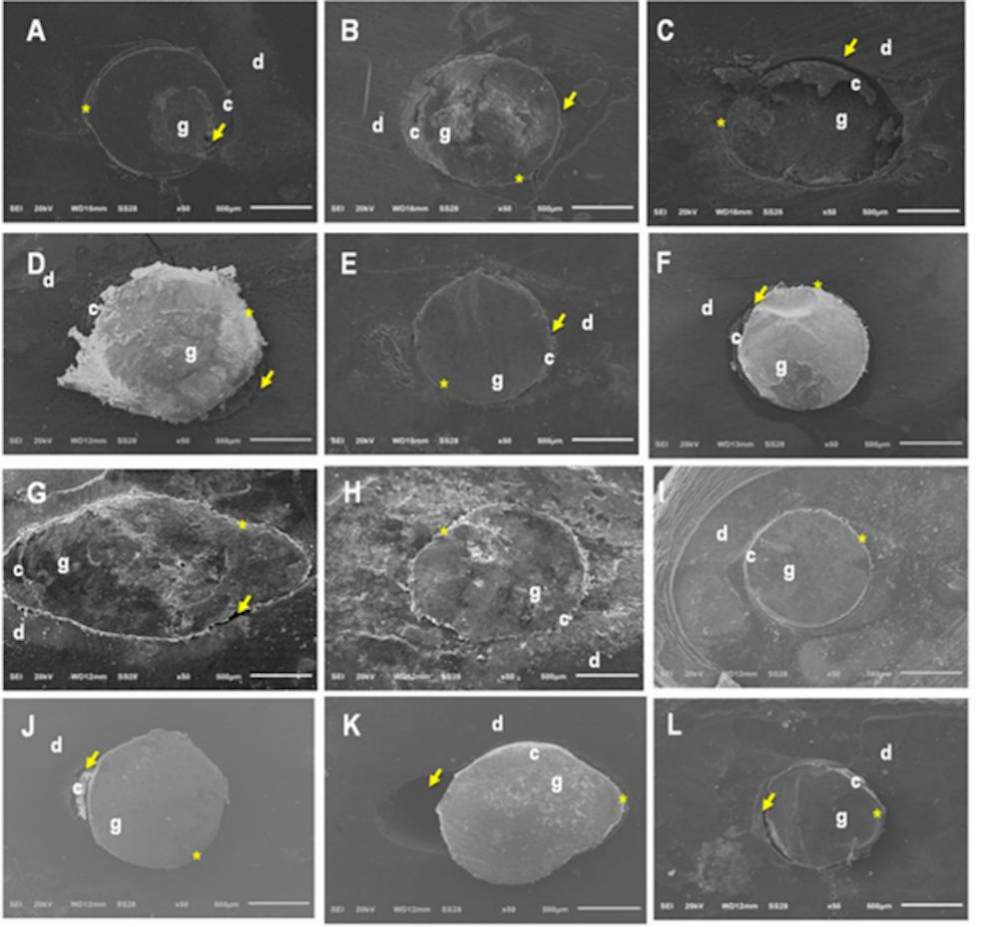
Photomicrographs of the adhesives interfaces of filling material to root dentin, obtained with the different filling techniques (50x). (A, B, C) Adhesive interface between the Bioceramic sealer when using the continuous wave condensation filling technique with Downpack at 4 mm, in the cervical middle thirds, and MTA barrier in the apical third, respectively. (D, E, F) Adhesive interface between Bioeramic sealer, Conventional gutta- percha point with Downpack at 4 mm, and root dentin in the cervical, middle, and apical thirds, respectively. (G, H, I) Adhesive interface between Bioeramic sealer, low-fusion gutta-percha point with Downpack at 4 mm, and root dentin in the cervical, middle, and apical thirds, respectively. (J, K, L) Adhesive interface between Bioceramic sealer, single low-fusion gutta-percha point, and root dentin in the cervical, middle, and apical thirds, respectively. d: dentin; c: endodontic sealer; g: gutta-percha; yellow asterisks: adaptation at the adhesive interface; yellow arrows; gaps at the adhesive interface.

Regarding the different evaluated thirds, in the cervical third, the low-fusion point group with the continuous wave technique showed higher temperature variation (8.5 ± 3.8) compared to the other groups (p<0.05). In the middle third, higher temperature variations were recorded in the low-fusion point group (9.5 ± 3.6) and the conventional points with continuous wave technique (6.2 ± 3.4), compared to the MTA group (0.9 ± 0.8) and the single-cone technique group (0.3 ± 0.2) (p<0.05). In the apical third, higher temperature variations were observed in the low-fusion point group (4.6 ± 2.4) and the conventional point group (3.0 ± 1.2), in comparison with the MTA group (0.3 ± 0.4) and the single-cone technique group (0.1 ± 0.2) (p<0.05). Qualitative SEM analysis (Figure 1) revealed areas of maladaptation (yellow arrows) and adaptation (yellow asterisks) at the adhesive interface between the filling material and root dentin in all experimental groups. In the cervical third (Figures 1A, 1D, 1G, 1J), areas of both adaptation and maladaptation of the adhesive interface were observed in all groups. Additionally, superimposition of the filling material onto the canal surface was noted in Figure 1D. In the middle (Figures 1B, 1E, 1H, 1K) and apical (1C, 1F, 1I, 1L) thirds, areas of both adaptation and maladaptation were observed, irrespective of the group.

In the analysis using scanning confocal microscopy (Figure 2), it was observed that regardless of the experimental group, there was greater maladjustment of the adhesive interface in the cervical (2A, 2D, 2G, 2J) and middle (2B, 2E, 2H, 2K) thirds, particularly in the polar areas. In the apical third (Figures 2C, 2F, 2I, 2L), adaptation at the adhesive interface was observed, with integrity of the filling margins, but some regions showed maladaptation.

Analysis of the confocal laser fluorescence microscopy revealed that regardless of the experimental group, there was no uniform penetration of the sealer into the adhesive interface (Figure 3) in the cervical (3A, 3D, 3G, 3J), middle (3B, 3E, 3H and 3K) and apical (3C, 3F, 3I, 3L) thirds, and there were more voids.

**Figure 2:**
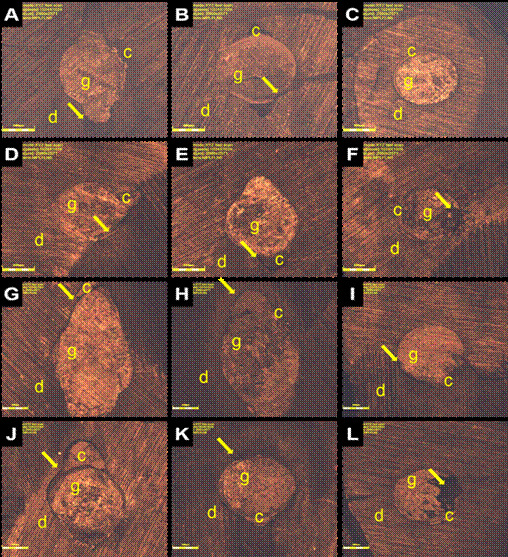
Photomicrographs of the adhesives interfaces of filling material to root dentin, obtained with the different filling techniques (50x). (A, B, C) Adhesive interface between the Bioceramic sealer when using the continuous wave condensation filling technique with Downpack at 4 mm, in the cervical middle thirds, and MTA barrier in the apical third, respectively. (D, E, F) Adhesive interface between Bioeramic sealer, Conventional gutta-percha point with Downpack at 4 mm, and root dentin in the cervical, middle, and apical thirds, respectively. (G, H, I) Adhesive interface between Bioeramic sealer, low-fusion gutta-percha point with Downpack at 4 mm, and root dentin in the cervical, middle, and apical thirds, respectively. (J, K, L- adhesive interface between Bioceramic sealer, single low-fusion gutta-percha point, and root dentin in the cervical, middle, and apical thirds, respectively. d - dentin, g - gutta-percha, sealer - sealer. Yellow arrows: maladaptation between the dentin, sealer, and gutta-percha.

**Figure 2:**
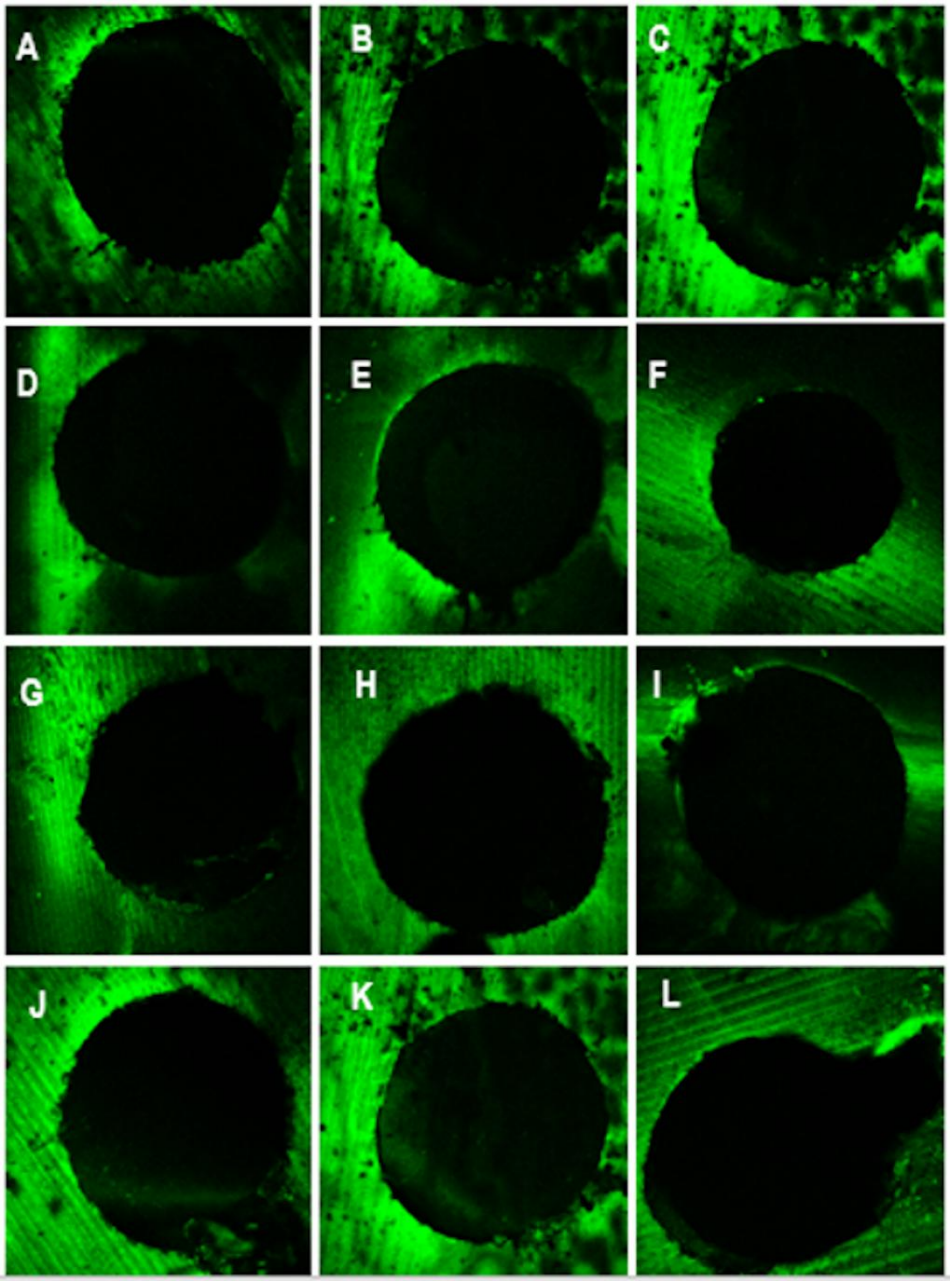
Photomicrographs of the adhesives interfaces of filling material to root dentin, obtained with the different filling techniques (50x). (A, B, C) Adhesive interface between the Bioceramic sealer when using the continuous wave condensation filling technique with Downpack at 4 mm, in the cervical middle thirds, and MTA barrier in the apical third, respectively. (D, E, F) Adhesive interface between Bioeramic sealer, Conventional gutta-percha point with Downpack at 4 mm, and root dentin in the cervical, middle, and apical thirds, respectively. (G, H, I) Adhesive interface between Bioeramic sealer, low-fusion gutta-percha point with Downpack at 4 mm, and root dentin in the cervical, middle, and apical thirds, respectively. (J, K, L- adhesive interface between Bioceramic sealer, single low-fusion gutta-percha point, and root dentin in the cervical, middle, and apical thirds, respectively. d - dentin, g - gutta-percha, sealer - sealer. Yellow arrows: maladaptation between the dentin, sealer, and gutta-percha.

## Discussion

Root canal treatment of necrotized immature teeth after traumatic injury is challenging due to the presence of an open apex, wide canals, and thin root dentin. Additionally, the lack of apical constriction requires careful control of the root canal filling length, as the absence of an apical stop can lead to extrusion of filling materials into the periapical tissues. As a result, it is recommended to use bioactive materials that promote interaction with dentin and the formation of an intermediate mineralized zone by physical-chemical interaction^17^. The study rejected the null hypothesis since the MTA apical barrier showed higher dislodgment resistance values and greater integrity of the adhesive interface.

In this study, higher dislodgment resistance values and a uniform adhesive interface were observed in the apical third of the teeth in which the apical MTA barrier was created, compared to the other groups (p<0.05). There was a predominance of mixed and cohesive failures involving dentin, attributed to MTA's hydration process^6^ ^,^ ^16^ ^,^ ^25^ ^,^ ^26^. The sealer particles promote volumetric expansion and reduce marginal gaps, thereby contributing to dislodgment resistance.

An MTA apical plug thickness of 4 mm in the apical third was chosen, as a thickness between 2 and 5 mm provides an apical seal with less possibility of marginal infiltration^10^ ^,^ ^11^. Harinkhere et al.^11^ observed less marginal infiltration with MTA compared to another bioceramic material (Biodentine), attributed to MTA's plasticity, which aids in adapting to the root walls and reduces microleakage. Additionally, the higher level of calcium ion release from calcium tungstate during the initial setting stage promotes greater dislodgment resistance and helps prevent bacterial infiltration^27^. Brito-Júnior et al. (2014)^28^ indicated that the association of an MTA apical plug with fiber posts constitutes a superior restorative strategy for immature teeth, as it promotes greater fracture resistance and a more homogeneous distribution of stresses along the root.

It is important to highlight that, apart from its ability to seal, when mineral trioxide aggregate (MTA) comes into direct contact with connective tissue, it generates calcium hydroxide and releases calcium ions, which help in cell attachment and growth. Furthermore, it encourages the differentiation and migration of cells responsible for producing hard tissues, resulting in the formation of hydroxyapatite (or carbonated apatite) on the material's surface. This process promotes the development of a mineralized barrier^6^.

In the current study, the groups that used the continuous wave filling technique showed the highest dislodgment resistance values compared to those using the single cone technique. This aligns with previous literature reporting lower dislodgment resistance with the single-cone technique ^29^ ^,^ ^30^. Bhandi et al.^31^ in a systematic review, noted that neither the lateral condensation nor the thermoplastic gutta-percha techniques resulted in a homogeneous filling without empty spaces when examined using micro-CT. However, evidence suggests that thermoplasticization techniques result in fewer voids, as thermoplasticized gutta-percha adapts to the polar areas of the canal, providing a gap-free filling^31^. This effect is attributed to the higher proportion of gutta-percha and a thinner sealer layer, which improves the adaptation of the root canal filling and explains the higher dislodgment resistance values. Conversely, the single cone technique has a lower proportion of gutta-percha in relation to sealer and a larger sealer line, which compromises interfacial adaptation and favors microleakage^19^. Additionally, the single-cone technique presents challenges in achieving apical sealing, potentially resulting in extrusion of the filling material into the periapical region^31^.

The dislodgment resistance values in the cervical and middle thirds were lower than those observed in the apical third, regardless of the group evaluated. No statistically significant differences were observed between the cervical and middle thirds. This finding may be attributed to morphological differences in dentin, as the apical third presents fewer and narrower dentinal tubules and a higher proportion of intertubular dentin compared with the middle and cervical thirds^33^ ^,^ ^34^. In addition, differences in dislodgment resistance among root thirds for different materials are likely related to their intrinsic material properties. The MTA barrier, used as a reparative material, favors interfacial adaptation, whereas bioceramic sealers present distinct characteristics such as flow and viscosity. These properties govern their physicochemical interaction with intertubular dentin, and variations in dentin morphology among root thirds may therefore influence the quality of interfacial adaptation.

In this study, SEM and confocal laser scanning fluorescence microscopy images showed the presence of bioceramic sealer in the cervical and middle thirds of the root canal. These images also revealed the prevalence of mixed and adhesive failures in the filling material, adhesive failures to dentin, and the presence of gaps ranging from 1 µm to 10 µm at the adhesive interface. Additionally, the sealer penetration into the adhesive interface was non-uniform, and there was a higher number of empty spaces present, irrespective of the group evaluated. These findings demonstrate the qualitative results of the study.

As regards heating during the filling techniques performed, we point out that the bioceramic sealer used in the present study maintains favorable physical-chemical properties even after exposure to heat, which makes it compatible with thermoplastic filling techniques^35^ ^,^ ^36^. Relative to temperature variations during different filling techniques, when comparing the thirds of the root canal it was observed that in the MTA group, low-fusion points group and conventional points group, both with continuous wave technique, the smallest temperature variations occurred in the apical third of the root canal when compared with the cervical and middle thirds, while in group of single cone technique no statistically significant difference was observed in the temperature variation between the different thirds of the root canal. This could be attributed to the depth of penetration of heat carriers and condensers, recommended in each of the techniques^37^. 

In the comparison between techniques and materials used, a higher increase in temperature was observed in the continuous wave condensation filling technique with the use of low-fusion points when compared with the technique with MTA barrier, conventional points, and the single cone technique. This result could be attributed to the fact that low-fusion points produce greater heat transmission across their surface^38^, leading to heat propagation to the root canal walls. Considering the foregoing, and given that the literature has shown that an increase of only 10 °C above body temperature (37 °C) can cause irreversible damage to supporting tissues, such as permanent vascular stasis, resorption, and even necrosis of bone tissue^18^ ^,^ ^39^ ^,^ ^40^, the use of the technique at a Downpack depth of 4 mm from the WL becomes questionable. Therefore, modifications in the technique are proposed, such as smaller penetration depths of the heat carrier^18^.

In this study, we initially performed a uniform distribution based on the two- and three-dimensional data obtained from cone beam computed tomography images. This was done to minimize the risk of bias ^41^ ^,^ ^42^. We used the push-out test methodology to evaluate the mechanical performance of the dislodgment resistance^20^ ^,^ ^21^ ^,^ ^23^ between the gutta-percha and the filling sealer along the root canal. Factors such as sample thickness, tip diameter, and root canal were standardized as they have a direct influence on the test results^43^ ^,^ ^44^. Additionally, we analyzed the failure pattern using a stereomicroscope and evaluated the adhesive interface with scanning electron microscopy and scanning confocal microscopy in order to visualize the endodontic filling sealer layer and identify possible voids in the adhesive interface^20^ ^,^ ^21^ ^,^ ^22^ ^,^ ^23^. We also used confocal laser scanning microscopy and incorporated the Fluo-3 dye into the calcium silicate-based sealer to assess the penetration of the filling sealer along the dentinal tubules. The Fluo-3 dye emits fluorescent light in green tones when exposed to calcium ions, and its intensity is proportional to the stability of the bonds formed^45^ ^,^ ^46^ ^,^ ^47^.

Based on the results obtained, it was observed that the MTA barrier remains an established protocol for root canal filling of teeth with open apex. It exhibited better results in dislodgment resistance, sealing ability, and quality of adhesive interface. Additionally, we analyzed the failure pattern using a stereomicroscope and assessed the adhesive interface by scanning electron microscopy and confocal microscopy to visualize the sealer layer and detect possible voids in the interface.

## Conclusion

In conclusion, for the filling of teeth with an open apex, the creation of an apical MTA barrier demonstrated the highest dislodgment resistance and superior adhesive interface quality. The use of a heat-compatible bioceramic sealer with the continuous wave condensation technique showed intermediate performance, regardless of the type of gutta-percha point used. In contrast, the single-cone technique resulted in the lowest dislodgment resistance values and adhesive interface quality.

## Data Availability

The research data are available upon request.
